# Development of a monoclonal antibody-based competitive ELISA as a surrogate assay for detecting neutralizing anti-interferon gamma autoantibodies in adult-onset immunodeficiency

**DOI:** 10.1371/journal.pone.0344451

**Published:** 2026-03-13

**Authors:** Kanokporn Sornsuwan, Kanyarat Thongheang, Ekkarat Wongsawat, Chatchai Tayapiwatana, Umpa Yasamut

**Affiliations:** 1 Office of Research Administration, Chiang Mai University, Chiang Mai, Thailand; 2 Center of Biomolecular Therapy and Diagnostic, Faculty of Associated Medical Sciences, Chiang Mai University, Chiang Mai, Thailand; 3 Division of Clinical Immunology, Department of Medical Technology, Faculty of Associated Medical Sciences, Chiang Mai University, Chiang Mai, Thailand; 4 Department of Medicine, Faculty of Medicine Siriraj Hospital, Mahidol University, Bangkok, Thailand; 5 Center of Innovative Immunodiagnostic Development, Department of Medical Technology, Faculty of Associated Medical Sciences, Chiang Mai University, Chiang Mai, Thailand; University of Miami, UNITED STATES OF AMERICA

## Abstract

Neutralizing anti-interferon gamma autoantibodies (nAIGAs) play a pivotal role in the pathogenesis of adult-onset immunodeficiency (AOID), predisposing affected individuals to severe opportunistic infections. Current detection of nAIGAs relies on cell-based assays requiring flow cytometric analysis, which limits routine clinical settings. Notably, nAIGAs recognizing the B27 epitope of interferon gamma (IFN-γ) exhibit strong neutralizing activity, underscoring the need for practical, epitope-specific detection methods for routine diagnostic. This study aimed to develop and validate a competitive enzyme-linked immunosorbent assay (cELISA) as a surrogate assay for detecting B27 epitope-targeting nAIGAs in plasma samples from patients with AOID. Plasma samples from patients with AOID (n = 40) and healthy controls (n = 40) were initially screened for AIGAs using an indirect ELISA. Neutralizing activity was confirmed using a cell-based assay measuring MHC class II expression on IFN-γ-stimulated THP-1 cells. The cELISA was subsequently developed and optimized to detect nAIGAs specific to the B27 epitope. ROC curve analysis was performed to assess the diagnostic performance of the assay. All AOID samples with detectable levels of AIGAs showed percentage inhibition above the cut-off in the cell-based assay, confirming the neutralizing activity of AIGAs. The developed cELISA detected nAIGAs in 36 of 40 AOID samples, with no false-positive results observed among healthy controls. ROC curve analysis indicated excellent diagnostic performance, yielding an area under curves (AUC) of 0.9684. Taken together, our findings indicate that the developed cELISA serves as a surrogate assay for detecting nAIGAs targeting the B27 epitope of IFN-γ. This assay represents a practical and scalable tool for AOID screening and may facilitate broader applications in clinical diagnostics.

## Introduction

Adult-onset immunodeficiency (AOID) is classified as a phenocopy of primary immunodeficiency, characterized by susceptibility to intramacrophagic pathogens [1]. It typically occurs in previously healthy, HIV-negative adults over 18 years of age, with a high prevalence reported in Asia—particularly in Thailand, Taiwan, and China [2–6]. AOID commonly presents with recurrent or disseminated infections caused by intracellular pathogens, among which non-tuberculous mycobacteria (NTM), *Talaromyces marneffei*, *Salmonella* species, and varicella-zoster virus (VZV) are the most frequently identified [4–6]. These infections often involve multiple organ systems and may be resistant to standard treatment [5–7]. A hallmark of AOID is the presence of nAIGAs. These autoantibodies block IFN-γ signaling, a critical pathway for macrophage activation and intracellular pathogen clearance [3,8–10].

Detection of AIGAs in patients with AOID involves two principal approaches: quantifying total AIGA levels using enzyme-linked immunosorbent assay (ELISA) and evaluating neutralizing activity through cell-based functional assays [10–13]. ELISA offers a scalable and high-throughput method for detecting binding antibodies but does not assess their functional capacity. In contrast, cell-based assays—such as those measuring IFN-γ-induced phosphorylated STAT1 (pSTAT1) or surface MHC class II expression—can determine the biological impact of AIGAs on downstream signaling [9,10]. Beyond conventional ELISA, AIGAs can also be detected using dried blood spots (DBS), which provide a simple and practical method for sample collection and transport. DBS-derived samples can be analyzed by multiplex particle-based assay to identify binding autoantibodies, and their neutralizing function can be further assessed by flow cytometry measuring IFN-γ–induced pSTAT1 [14]. Despite their clinical relevance and strong association with disease severity, detection of nAIGAs remains limited in routine laboratories due to the technical complexity and need for specialized equipment. This highlights the need for practical, accessible diagnostic platforms capable of reliably identifying nAIGAs in patient samples.

Competitive ELISA (cELISA) is a versatile platform for detecting antibodies that target functionally relevant epitopes, particularly neutralizing antibodies (nAbs), by assessing their ability to compete with reference antibodies for antigen binding. This indirect approach enables estimation of neutralizing potential without the need for cell-based systems. Surrogate antibody-based cELISAs further enhance specificity by incorporating well-characterized monoclonal antibodies with defined neutralizing activity—such as those used in SARS-CoV-2 assays to evaluate antibody-mediated blockade of the Spike–ACE2 interaction [15]. In this context, a cELISA was developed using monoclonal antibody 6B211, which targets the same epitope on the C-strain E2 protein as vaccine-induced nAbs, enabling its use as a surrogate for virus neutralization testing (VNT) in post-vaccination monitoring of classical swine fever (CSF) vaccines [16]. Although cELISA does not directly assess downstream biological functions, it provides a scalable, high-throughput alternative for nAb screening in clinical and research settings, particularly where access to cell-based assays or specialized equipment is limited.

In this study, cELISA was developed to detect nAIGAs, using the mouse anti-IFN-γ monoclonal antibody (clone B27, B27 mAb) as a surrogate competitor. This antibody was chosen because it exhibits strong neutralizing activity, and AIGAs targeting its epitope are frequently observed in patients with AOID [9,17]. Unlike indirect ELISA, which quantifies total binding antibodies without assessing function, cELISA directly measures competition for IFN-γ binding, thereby providing greater functional relevance for clinical diagnosis. To validate performance, cELISA was compared with a cell-based assay measuring IFN-γ–induced surface MHC class II expression. Although both pSTAT1 and MHC class II assays are considered functional gold standards, MHC class II was selected here because it is technically simpler and more practical for large-scale sample processing. These considerations highlight the potential of cELISA as a scalable and accessible approach for nAIGA screening, positioning it as a valuable tool for both clinical application and research investigation.

## Materials and methods

### Recombinant human IFN-γ production

Recombinant human interferon-γ (rhIFN-γ) was produced in *Escherichia coli* (*E. coli*) strain BL21(DE3), as previously described [9]. Briefly, the pET21a plasmid encoding rhIFN-γ with a C-terminal hexahistidine tag (His6 tag) was transformed into *E. coli* BL21(DE3), and the cells were cultured in Super Broth supplemented with 1% glucose and 100 µg/mL ampicillin at 37°C with shaking at 200 rpm. When the optical density at 600 nm (OD_600_) reached 0.6–0.8, protein expression was induced with 1 mM isopropyl β-D-1-thiogalactopyranoside (IPTG), followed by incubation for an additional 16 hours at 30°C. Induced cells were harvested and lysed by sonication, and the lysate was clarified by centrifugation at 12,000 × g for 30 minutes at 4°C. The recombinant protein was purified by affinity chromatography using a HisTrap column on an ÄKTA Prime Plus system (GE Healthcare, Piscataway, NJ).

### Plasma samples

This retrospective study utilized residual plasma samples, stored at −80°C, obtained from a previous study [18]. A total of 40 samples from AOID patients (17 males, 23 females; aged 25–68 years)—all HIV-negative, AIGA-positive (AIGA^+^), and presenting with at least one opportunistic infection, including NTM, *Salmonella* spp., *Mycobacterium tuberculosis*, *Talaromyces marneffei*, or varicella-zoster virus (VZV)—were examined, together with 40 healthy controls (HC) (11 males, 29 females; aged 24–57 years) who had no evidence of infection and were confirmed to be negative for AIGAs. The levels and neutralizing activity of AIGAs were analyzed, with cut-off values for each assay determined as the mean plus two standard deviations (SD) of the HC samples. Ethical approval for the use of these samples was obtained from the Siriraj Institutional Review Board (SIRB Protocol No. 490/2562, EC2). Archived plasma samples were accessed for research purposes during the ethically approved period from 2 October 2019–1 October 2023. All data were fully anonymized prior to investigator access, ensuring that no identifiable patient information was available during the study.

### Indirect ELISA for detection of AIGA levels

Microwells were coated with 50 µL of rhIFN-γ at a concentration of 2.5 µg/mL in bicarbonate buffer and incubated overnight at 4°C in a humidified chamber. The coated wells were washed four times with 0.05% Tween-20 in phosphate-buffered saline (PBS) (washing buffer), followed by blocking with 200 µL of 2% skimmed milk in PBS (blocking buffer) at room temperature for 1 hour. After two washes, 50 µL of plasma (1:500 dilution in blocking buffer) was added and incubated for 1 hour. The wells were then washed four times, and 50 µL of horseradish peroxidase (HRP)-conjugated rabbit anti-human IgG antibody (1:3,000 dilution; SeraCare, Milford, MA) was added and incubated for 1 hour. After four additional washes, the reaction was developed using TMB chromogen substrate (SeraCare, Milford, MA) and stopped after 10 minutes with 1 N HCl. The optical density at 450 nm (OD₄₅₀) was measured using a microplate reader.

### Cell-based functional assay for detection of nAIGAs

Neutralizing activity of AIGAs in plasma samples was assessed using a cell-based functional assay with the monocytic THP-1 cell line (ATCC). The Plasma samples were diluted 1:50 and pre-incubated at a 1:1 volume ratio with 40 ng/mL rhIFN-γ at room temperature for 1 hour. After incubation, 200 µL of the plasma–rhIFN-γ mixture was added to 4 × 10⁵ THP-1 cells suspended in 200 µL of complete RPMI medium, resulting in a final plasma dilution of 1:200 and a rhIFN-γ concentration of 10 ng/mL. The cells were then incubated at 37°C in a humidified 5% CO₂ atmosphere for 24 hours. Following incubation, cells were harvested for surface staining of MHC class II molecules. The cells were washed twice with PBS and subsequently blocked with 50 µL of 10% heat-inactivated human AB serum in PBS for 30 minutes on ice. Subsequently, 2.5 µL of FITC-conjugated anti-human HLA-DR and HLA-DP antibodies, or an isotype-matched control antibody (ImmunoTools, NI, DE), was added and incubated for 30 minutes on ice. After three washes with FACS buffer (2% heat-inactivated fetal bovine serum (HI-FBS), 0.5 mM EDTA, 0.01% NaN_3_ in PBS), cells were resuspended in 1% paraformaldehyde in PBS (fixation buffer). MHC class II–positive cells were analyzed using a BD Accuri^TM^ C6 Plus flow cytometer (BD Biosciences, Bergen, NJ, USA), and data were processed using FlowJo software. The stimulation index (SI) was defined as the ratio of the mean fluorescence intensity (MFI) of IFN-γ–stimulated cells to that of unstimulated cells. The percent inhibition (% inhibition) was then calculated from the SI as follows: the SI of IFN-γ–treated cells without plasma was subtracted from the SI of IFN-γ–treated cells with plasma, divided by the SI of IFN-γ–treated cells without plasma, and multiplied by 100.

### HRP labeling of mouse B27 mAb

Conjugation of the B27 mAb to HRP was performed using the LYNX Rapid HRP Antibody Conjugation Kit (Bio-Rad Laboratories Ltd., Watford, UK), in accordance with the manufacturer’s instructions. Briefly, 10 µL of modifier reagent was mixed with 100 µL of B27 mAb (2 mg/mL) and added to 100 µg of lyophilized HRP. The conjugation reaction was performed at a molar ratio of 1:2 (antibody: HRP). The mixture was incubated at room temperature for 3 hours. Following incubation, 10 µL of quencher reagent was added, and the solution was allowed to stand for 30 minutes. The HRP-conjugated B27 mAb (B27 mAb-HRP) was mixed with glycerol to a final concentration of 50%, aliquoted, and stored at −20°C.

### Optimization of B27 mAb-HRP and IFN-γ concentration for cELISA

Microwells were coated with 50 µL of rhIFN-γ at varying concentrations (0, 0.625, 1.25, 2.5, and 5 µg/mL) in bicarbonate buffer (pH 9.6) and incubated overnight at 4°C in a humidified chamber. After incubation, wells were washed four times with washing buffer and subsequently blocked with 200 µL of blocking buffer at room temperature for 1 hour. The wells were then washed twice, followed by the addition of 50 µL of B27 mAb-HRP, serially diluted two-fold from 1:2,500–1:40,000, and incubated for 1 hour. After washing, the reaction was developed using TMB chromogen substrate (SeraCare, Milford, MA) and stopped by adding 1 N HCl after 10 minutes. The OD₄₅₀ was measured using a microplate reader.

### cELISA for detection of nAIGAs

Microwells were coated with 50 µL of rhIFN-γ at a concentration of 2.5 µg/mL in bicarbonate buffer (pH 9.6) and incubated overnight at 4°C in a humidified chamber. Following incubation, wells were washed four times with washing buffer and blocked with 200 µL of blocking buffer at room temperature for 1 hour. After two additional washes, 50 µL of a mixture containing serum (final dilution 1:100) and B27 mAb-HRP (final dilution 1:2,500) in blocking buffer was added to each well and incubated for 1 hour. Following four washes, the reaction was developed using TMB chromogen substrate (SeraCare, Milford, MA) and stopped after 10 minutes by adding 1 N HCl. Each sample was assayed in duplicate, and the OD₄₅₀ was measured using a microplate reader. The mean OD₄₅₀ value was used to calculate the percentage inhibition (% inhibition), defined as: [(OD₄₅₀ of B27 mAb-HRP without plasma − OD₄₅₀ of B27 mAb-HRP with plasma)/ OD₄₅₀ of B27 mAb-HRP without plasma] × 100.

### Receiver operating characteristic (ROC) analysis

Assay performance was evaluated using ROC analysis. ROC curves were generated using GraphPad Prism 9, and sensitivity and specificity were calculated at various cutoff values. Corresponding 95% confidence intervals were determined to assess the diagnostic accuracy of the assay. The Youden’s index (*J*) was employed to determine the optimal cut-off value that maximizes both sensitivity and specificity of assay. This index is calculated as: *J* = sensitivity+specificity-1.

### Statistical analysis

Statistical differences in OD₄₅₀ values from indirect ELISA, % inhibition from the cell‑based assay, and % inhibition from cELISA between HC and AIGA⁺ groups were determined using the Mann–Whitney U test. Statistical significance was defined as p ≤ 0.05.

## Results

### Evaluation of AIGA levels and nAIGA activity in plasma samples

**Fig 1:**
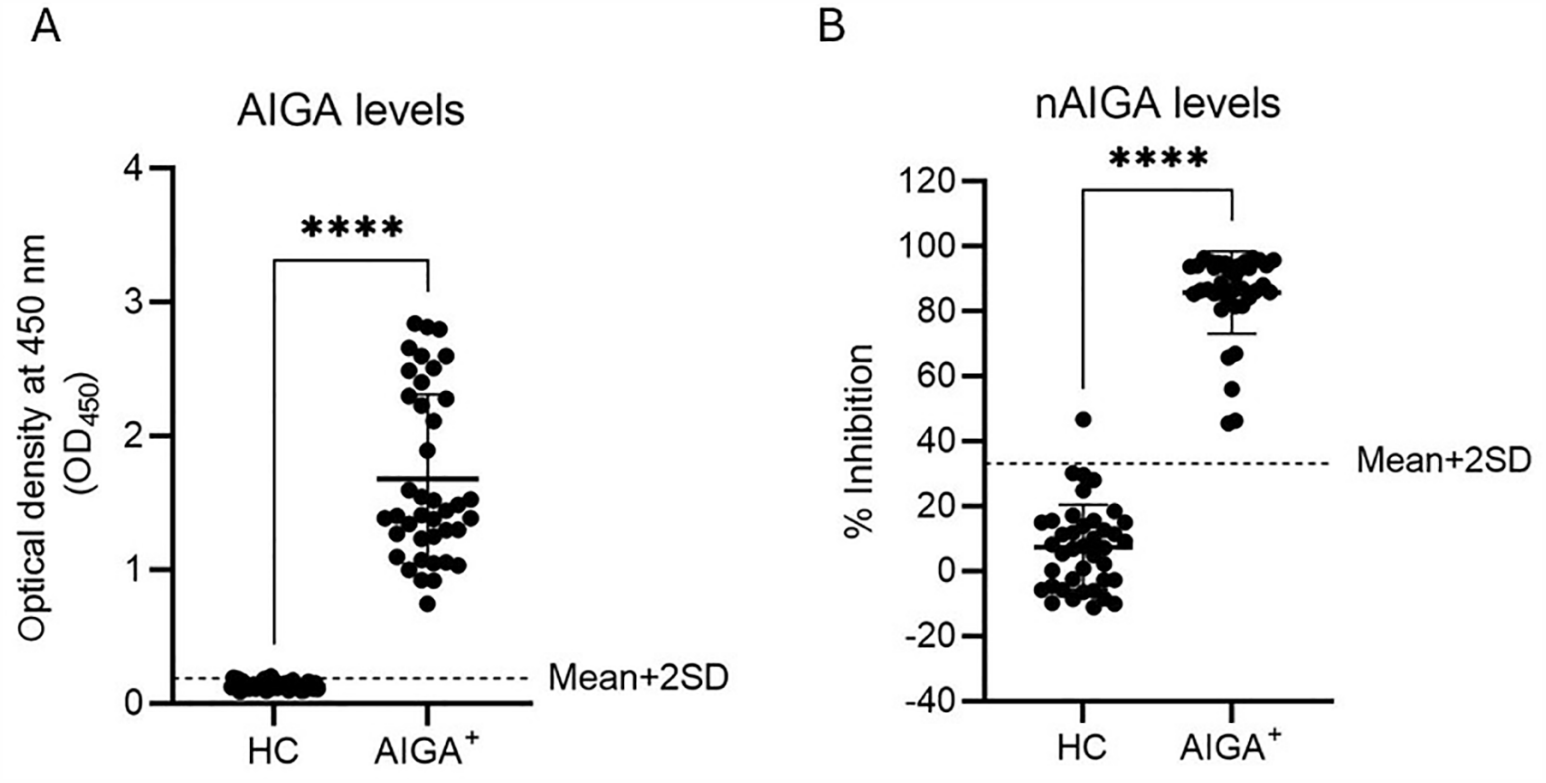
Detection of AIGA and nAIGA levels in plasma samples. **(A)** Levels of AIGAs in plasma from AOID patients (n = 40) and healthy individuals (n = 40) were measured using indirect ELISA. Cut-off value for indirect ELISA was defined as the mean OD₄₅₀ of HC samples plus two standard deviations. **(B)** The nAIGA levels in these samples were assessed using a cell-based functional assay in THP-1 cells. Cut-off value for this assay was defined as the mean % inhibition of HC samples plus two standard deviations. HC = HC samples; AIGA^+^ = AIGA-positive samples. The statistical difference between two groups was determined by Mann-Whitney U test (**** *p* < 0.0001).

The levels of AIGAs in plasma samples from AOID patients and healthy controls were evaluated using indirect ELISA (Fig 1A). The cut-off value for the indirect ELISA was set at OD₄₅₀ = 0.189, calculated as the mean OD₄₅₀ of HC samples plus two standard deviations (mean + 2SD = 0.133 + 0.056). All patient samples exhibited markedly elevated AIGA levels (mean ± SD = 1.68 ± 0.633), exceeding the established cut-off. Indirect ELISA results for AIGA^+^ and HC samples are summarized in S1 Data.

To assess the functional activity of nAIGAs, a cell‑based assay was performed to measure inhibition of IFN‑γ‑induced MHC class II upregulation in THP‑1 cells (Fig 1B). The flow cytometry gating strategy used to determine MHC class II expression in THP‑1 cells is shown in S1 Data. The cut‑off for the cell‑based assay was defined as 33.34% inhibition, calculated from the mean % inhibition observed in HC samples plus two standard deviations (mean + 2SD = 7.14 + 26.2). All 40 patient samples showed % inhibition above the assay cut‑off (mean ± SD = 85.8 ± 12.7). Of these, 35 demonstrated high neutralizing activity (>80% inhibition), while the remaining 5 exhibited moderate activity above the cut‑off but below 80% inhibition. % inhibition values from the cell‑based assay for AIGA+ and HC samples are summarized in S1 Data.

### Design and optimization of cELISA for detecting nAIGAs

**Fig 2:**
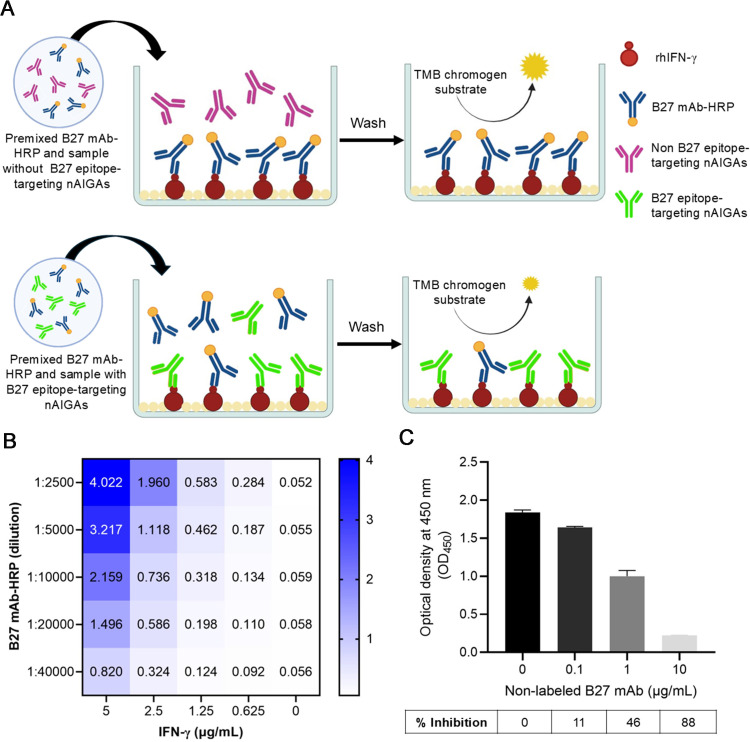
Optimization of cELISA for detecting nAIGAs. **(A)** Schematic representation of the cELISA principle for detecting B27 epitope‑targeting nAIGAs in plasma. Plasma samples were preincubated with B27 mAb-HRP and subsequently applied to microwells coated with rhIFN‑γ. In the absence of B27 epitope‑targeting nAIGAs, B27 mAb-HRP binding to rhIFN‑γ was unimpeded, producing a strong signal (upper panel). Conversely, in the presence of B27 epitope‑targeting nAIGAs, competitive inhibition prevented B27 mAb binding to rhIFN‑γ, resulting in diminished signal following substrate addition (lower panel). **(B)** Heatmap of OD₄₅₀ signals generated by serial dilutions of B27 mAb-HRP binding to microwells coated with increasing concentrations of rhIFN‑γ. The mean OD₄₅₀ values of duplicate wells from two independent experiments were used to construct the heatmap. **(C)** Validation of the optimized condition using non‑labeled B27 mAb (0–10 µg/mL) competing with B27 mAb-HRP (1:2,500 dilution). The bar graph shows the mean ± SD from duplicate wells.

A cELISA was developed to detect B27 epitope targeting nAIGAs. The assay principle is illustrated in Fig 2A. To optimize assay performance, various concentrations of rhIFN-γ and B27 mAb-HRP were systematically evaluated. As shown in the ELISA optimization heatmap (Fig 2B), the highest OD_450_ values were observed at 5 µg/mL rhIFN-γ, with the strongest signal at a 1:2,500 dilution of HRP-conjugated B27 mAb (OD₄₅₀ ≈ 4.022). A 1:5,000 dilution also produced a robust signal (OD₄₅₀ ≈ 3.217), although both dilutions showed signs of signal saturation. Signal intensity declined with increasing antibody dilution and decreasing rhIFN-γ concentration. At 0 µg/mL rhIFN-γ, OD_450_ values remained near baseline, confirming the assay’s specificity. Based on these results, the optimal condition was determined to be 2.5 µg/mL rhIFN-γ coating and a 1:2,500 dilution of B27 mAb–HRP, providing a strong, quantifiable signal while avoiding saturation.

To further validate the competitive format of the assay, non‑labeled B27 mAb (0–10 µg/mL) was tested for its ability to compete with HRP‑conjugated B27 mAb. The non-labeled B27 mAb was pre-mixed with B27 mAb-HRP (1:2,500 dilution) and added to rhIFN-γ-coated microwells. As shown in Fig 2C, OD_450_ values decreased in a dose-dependent manner, with percentage inhibition values of 0%, 11%, 46%, and 88% for 0, 0.1, 1, and 10 µg/mL of non-labeled B27 mAb, respectively. These results confirm the assay’s ability to detect AIGAs that specifically compete for the B27 epitope.

To assess assay specificity, the cELISA was tested against IL‑17A as an irrelevant antigen (S1 Data). B27 mAb-HRP produced clear signals in IFN‑γ–coated wells, whereas signals in IL‑17A–coated wells remained at background levels. Similarly, both AIGA⁺ and HC plasma samples generated detectable signals in IFN‑γ–coated wells but not in IL‑17A–coated wells. These findings demonstrate that the assay is highly specific to IFN‑γ and supports its suitability for diagnostic and translational applications.

### Neutralizing AIGAs detection in patient plasma using cELISA

**Fig 3:**
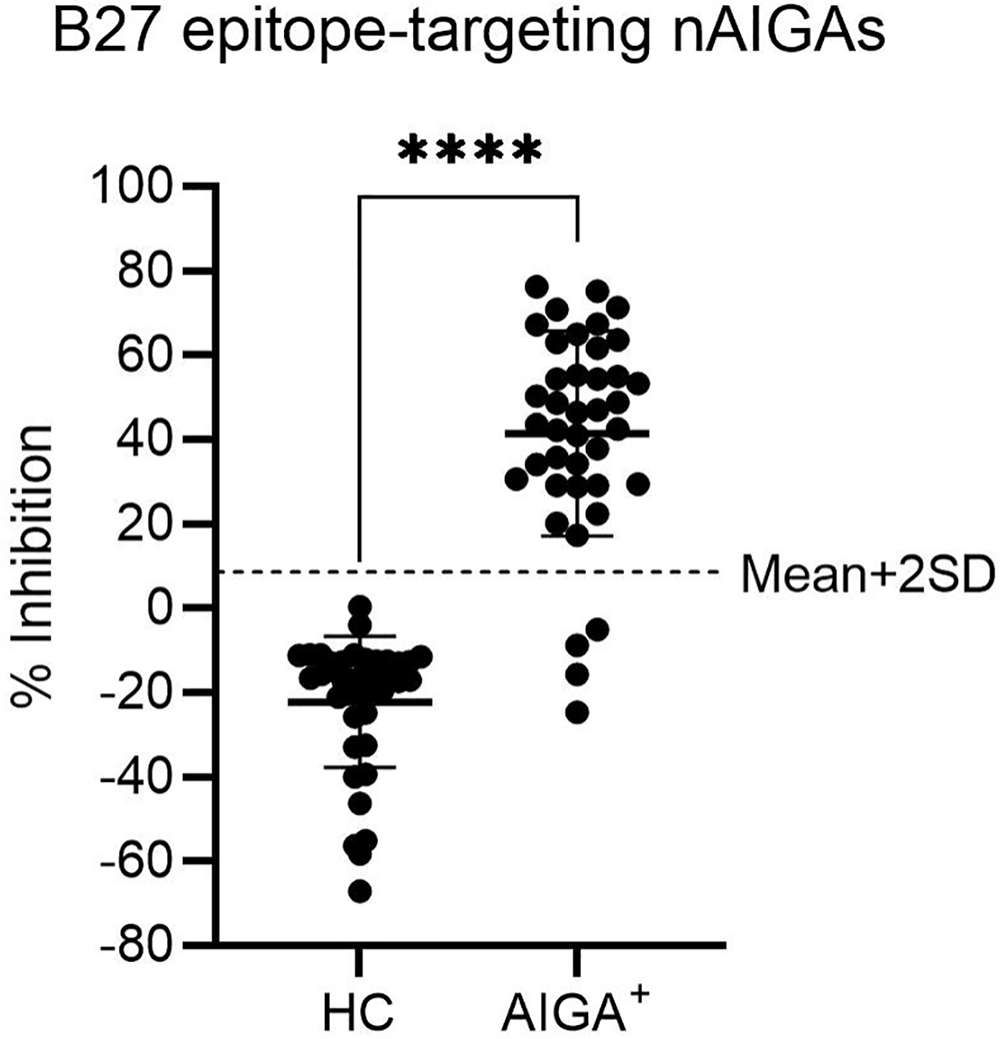
Detection of B27 epitope-targeting nAIGAs using cELISA. The inhibition levels of B27 epitope-targeting nAIGAs in plasma samples from HCs (n = 40) and AOID patients (n = 40) were assessed using cELISA. Each sample was assayed in duplicate, and the mean OD_450_ was used to calculate the % inhibition. The assay cut-off was defined as 8.89% inhibition, calculated from the mean percentage inhibition observed in HC samples (−22.3%) plus two standard deviations (2SD = 31.2%). HC = healthy control samples; AIGA⁺ = AIGA-positive samples. The statistical difference between AIGA+ and HC was determined using Mann-Whitney U test. **** *p* ≤ 0.0001.

To evaluate the presence of nAIGAs, plasma samples from HCs and patients were analyzed using the optimized cELISA, based on the detection principle shown in Fig. 2A. The cut-off value for nAIGAs positivity was defined as 8.89% inhibition, calculated from the mean inhibition observed in HC samples plus two standard deviations (mean + 2SD = −22.3 + 31.2). All HC samples exhibited inhibition levels below assay cut-off (mean ± SD = −22.3 ± 15.6) (Fig 3). The AIGA⁺ samples showed variable inhibition levels in the cELISA, with a mean ± SD of 41.4 ± 24.3%. Among the 40 patient samples tested, 36 showed inhibition levels exceeding the cut-off, indicating the presence of B27 epitope targeting nAIGAs. The remaining four samples exhibited inhibition levels below the defined threshold, suggesting that these individuals may harbor AIGAs targeting epitopes distinct from the B27-recognized region, which do not interfere with B27 mAb binding in this assay format. % inhibition values from cELISA for AIGA⁺ and HC samples are summarized in S1 Data.

### Assay performance determination using ROC analysis

**Fig 4:**
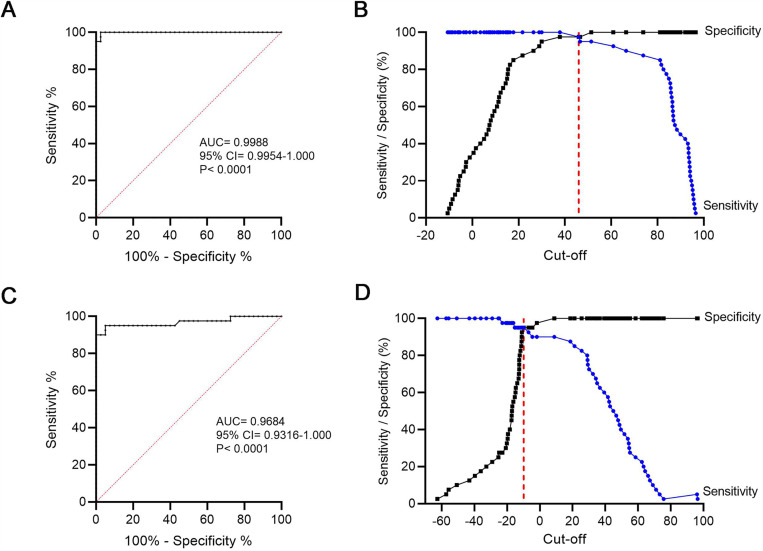
ROC analysis of assays for detecting nAIGAs. ROC analysis was performed to evaluate the diagnostic performance of two assays. The AUC provides a measure of diagnostic accuracy, with values between 0.800 and 1.000 considered indicative of good to excellent performance. Sensitivity and specificity were also plotted against cut-off values to determine the optimal threshold for each assay. **(A)** ROC curve and (B) sensitivity–specificity versus cut-off plot of the cell-based functional assay. **(C)** ROC curve and (D) sensitivity–specificity versus cut-off plot of cELISA. The red dashed line indicates the optimal cut-off that maximizes both sensitivity and specificity.

The performance of two assays for detecting nAIGAs—a cell-based functional assay and a cELISA—was evaluated using receiver operating characteristic (ROC) analysis. The ROC datasets for each assay are summarized in S1 Data. The cell-based functional assay yielded an area under the curve (AUC) of 0.9988 (95% CI: 0.9954–1.000, P < 0.0001), indicating perfect sensitivity and specificity with no false-positive or false-negative results (Fig 4A). ROC analysis suggested the optimal cut-off at 45.95% inhibition yielding 97.5% sensitivity and 97.5% specificity with Youden’s index (*J*) at 1.00 (Fig 4B). At a lower cut-off of 37.85% inhibition, the sensitivity increases to 100% while retaining specificity at 97.5% (J = 0.975). The specificity of test aligns with the observation that one of forty healthy donors was classified as positive for nAIGAs.

In comparison, the ROC curve for the cELISA (Fig 4C) demonstrated a high AUC of 0.9684 (95% CI: 0.9316–1.000, P < 0.0001), indicating strong—though slightly lower—diagnostic performance relative to the cell-based assay. Based on ROC analysis, −9.93% inhibition yielded 95% sensitivity and 95% specificity (J = 0.90) (Fig 4D). Increasing the cut-off value markedly improved assay specificity whereas sensitivity declined. These findings suggest that while the cELISA is highly specific, its sensitivity is limited to detecting nAIGAs that compete for the B27-recognized epitope, and may not detect antibodies targeting other regions of IFN-γ. Additionally, ROC analysis of the indirect ELISA was performed to assess assay performance (S1 Data), yielding an AUC of 1.000 (95% CI: 0.932–1.000; P < 0.0001).

### Assay correlation between cELISA and cell-based functional assay

**Fig 5:**
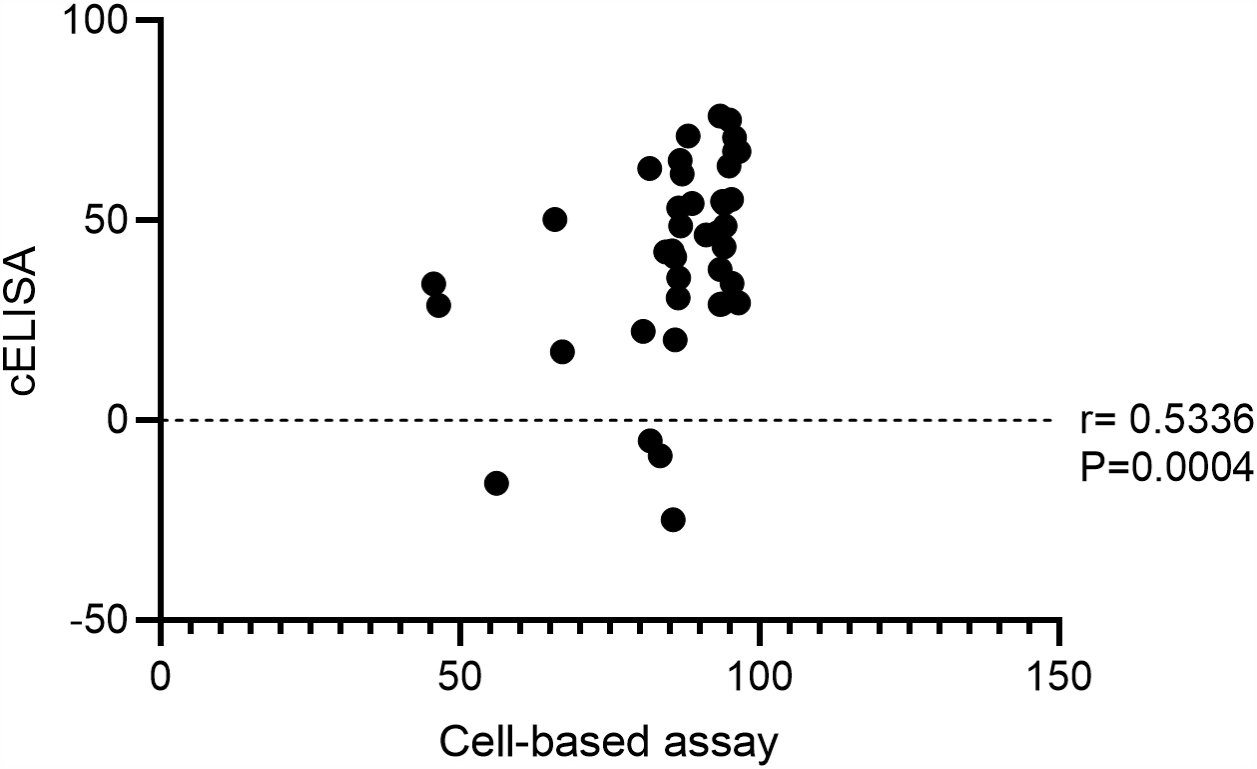
Correlation between cELISA and the cell-based assay. The association between percentage inhibition values obtained from the two methods was evaluated using Spearman’s correlation coefficient. A dotted line indicates zero inhibition as a reference. The correlation coefficient (r) and corresponding P-value are displayed on the plot.

The assay correlation between cELISA and cell-based functional assay was analyzed (Fig 5). A moderate positive correlation was observed between two assays (r = 0.5336, P = 0.0004). Overall, higher responses in the cell-based assay were associated with increased cELISA values. The partial disagreement observed between two assay platforms reflecting the discordant results from 4 samples with a measurable cell-based activity but low or negative cELISA values.

## Discussion

In this study, we developed a cELISA specifically designed to detect AIGAs targeting the B27 epitope, which has been reported to mediate potent neutralizing activity in patients with AOID [9]. Our results indicate that these antibodies may function as biomarkers of disease activity, underscoring the value of epitope-specific detection for identifying neutralizing antibodies. Accordingly, the assay incorporates the well-characterized B27 mAb with established neutralizing capacity [19] and is intended to serve as a surrogate for cell-based neutralization assays. Similar epitope‑focused cELISA platforms have proven effective in other contexts, including COVID‑19 convalescent plasma [15] and detection of neutralizing antibodies against the C‑strain E2 antigen in pigs [16], supporting the broader utility of this approach.

In our cohorts, the cell‑based functional assay achieved an AUC of 0.9988, reaffirming its role as the gold standard due to superior sensitivity and direct functional relevance. Nevertheless, this assay remains technically demanding and time‑intensive. By comparison, the B27‑focused cELISA identified 36 of 40 AOID patients as positive, yielding 90% sensitivity and 100% specificity (AUC 0.9684). Although slightly less sensitive, the absence of false positives underscores its diagnostic robustness and cost‑effectiveness, highlighting its potential for broader implementation. Importantly, the determination of cut‑off values should be guided by the intended purpose of the assay, as different applications may prioritize sensitivity or specificity. In addition, interpretation of positive results requires caution, as they indicate the presence of B27 epitope‑targeting nAIGAs but do not exclude the possibility of other nAIGA clones.

Of note, one HC sample exhibited neutralizing activity in the cell‑based assay while remaining negative in both indirect ELISA and cELISA. The observed reduction in MHC II expression, reflected by the percentage of inhibition, indicates that certain factors interfered with IFN‑γ–mediated signaling rather than representing true pathogenic autoantibodies. Such discordance is most plausibly attributable to serum matrix effects or assay interference, which can alter cellular responsiveness to IFN‑γ stimulation. Moreover, because cut‑off values were defined as mean ± 2 SD of HC samples, ~ 2.5% of healthy individuals are statistically expected to exceed the threshold [20], rendering this single HC finding consistent with the anticipated false‑positive rate. Collectively, these observations underscore the importance of interpreting binding and functional assays in parallel to reliably distinguish true pathogenic autoantibodies from assay‑related variability.

The nAIGAs found in AOID patients are known to interfere with IFN-γ signaling by blocking receptor binding and disrupting IFN-γR1–IFN-γR2 heterodimerization [21]. To overcome the limitation of relying on a single mAb, incorporating additional clones in parallel may broaden epitope coverage and enhance detection sensitivity. For instance, the anti-IFN-γ mAb clone A9 targets the C-terminal epitope of IFN-γ, a region previously reported to be bound by AIGAs in AOID patients [9,22]. Another clone, NI-0501—a fully human mAb directed against IFN-γ—exhibits neutralizing activity by preventing receptor dimerization [23]. Given that the sensitivity of our cELISA was 90% (with 4 out of 40 patients testing negative for B27-epitope–specific nAIGAs), the inclusion of additional mAbs may improve assay performance and expand the diagnostic utility of epitope-focused platforms.

Apart from AOID, AIGAs have also been associated with chronic infections caused by intracellular pathogens such as mycobacteria [24]. More recently, neutralizing anti‑IFN‑γ IgG has been reported in patients with systemic lupus erythematosus (SLE), particularly those experiencing severe infections, where elevated titers correlated with disease activity and impaired IFN‑γ‑induced STAT1 phosphorylation [25]. These findings indicate that the presence of AIGAs is not limited to AOID but may also contribute to increased infection susceptibility in autoimmune diseases. Recognition of such autoantibodies is clinically important, as it may inform the use of adjunctive immunomodulatory strategies in combination with appropriate antimicrobial therapy.

Key limitations of this study include the modest sample size and limited HC cohort. Future work should validate performance in larger, diverse populations and incorporate additional epitopes. Longitudinal and multi‑center studies, together with exploration of broader cytokine autoantibody panels, will be essential for establishing standardized protocols and integrating cELISA into comprehensive immunodiagnostic frameworks.

## Conclusion

The developed epitope‑focused cELISA demonstrates the ability to detect clinically relevant nAIGAs in patients with AOID. By targeting functionally relevant epitopes, this assay serves as a surrogate functional assay, providing a scalable and specific tool for nAIGA detection. With further validation, its application has the potential to improve diagnostic workflows, support disease monitoring, and contribute to therapeutic decision‑making in AIGA‑associated immunodeficiency.

## Supporting information

S1 Data S1 Table. Dataset of AIGA^+^ and HC plasma samples tested by indirect ELISA, cell-based assay and cELISA. S2 Table. Dataset for sensitivity and specificity analysis of cell-based assay and cELISA using ROC analysis. S1 Fig. Flow cytometry gating strategy for determination of MHC class II expression in THP-1 cells. A representative gating strategy is shown for THP-1 cells under three conditions: no plasma, healthy control (HC), and AIGA-positive (AIGA⁺). THP-1 cells were first identified based on forward scatter height (FSC-H) and side scatter height (SSC-H) properties to exclude debris. Doublets were then removed by FSC-A versus FSC-H gating to define singlets. MHC class II–positive cells were subsequently identified based on FITC fluorescence intensity (Comp-FL1-H). The mean fluorescence intensity (MFI) of MHC class II–positive cells was used to calculate percentage inhibition. S1 Experiment. Assay specificity validation of cELISA. S1 Text. Assay performance of indirect ELISA.
(ZIP)
