# Supplementary material for: Development of a monoclonal antibody-based competitive ELISA as a surrogate assay for detecting neutralizing anti-interferon gamma autoantibodies in adult-onset immunodeficiency
Source: PLoS One. 2026 Mar 13;21(3):e0344451. doi: 10.1371/journal.pone.0344451 (PMC12987466; doi:10.1371/journal.pone.0344451)
Supplement: S1 Data — S2 Table. Dataset for sensitivity and specificity analysis of cell-based assay and cELISA using ROC analysis. S1 Fig. Flow cytometry gating strategy for determination of MHC class II expression in THP-1 cells. A representative gating strategy is shown for THP-1 cells under three conditions: no plasma, healthy control (HC), and AIGA-positive (AIGA⁺). THP-1 cells were first identified based on forward scatter height (FSC-H) and side scatter height (SSC-H) properties to exclude debris. Doublets were then removed by FSC-A versus FSC-H gating to define singlets. MHC class II–positive cells were subsequently identified based on FITC fluorescence intensity (Comp-FL1-H). The mean fluorescence intensity (MFI) of MHC class II–positive cells was used to calculate percentage inhibition. S1 Experiment. Assay specificity validation of cELISA. S1 Text. Assay performance of indirect ELISA. (ZIP) [file pone.0344451.s001.zip › Supporting Information/S1 Experiment.docx]

**S1 Experiment.** **Assay specificity validation of cELISA.**

**Materials and methods**

To validate the specificity of the developed cELISA, B27 mAb-HRP, with or without plasma samples, was tested in parallel on microwells coated with recombinant human IFN‑γ (rhIFN‑γ) or rhIL‑17A, used as an irrelevant cytokine. Microwells were coated with 50 µL of rhIFN‑γ or rhIL‑17A (Sinobiological, Beijing, China) at 2.5 µg/mL in bicarbonate buffer, pH 9.6 and incubated overnight at 4 °C in a humidified chamber. Following incubation, wells were washed four times with PBS containing 0.05% Tween‑20 (washing buffer) and blocked with 200 µL of 2% skimmed milk in PBS (blocking buffer) at room temperature for 1 hour. After two additional washes, 50 µL of a mixture containing representative HC (n = 5) or AIGA⁺ (n = 5) plasma samples (final dilution 1:100) and B27 mAb-HRP (final dilution 1:2,500) in blocking buffer was added to each well and incubated for 1 hour. After four washes, the reaction was developed with 3,3′,5,5′‑tetramethylbenzidine (TMB) substrate (SeraCare, MA, USA) for 10 minutes and terminated with 1 N HCl. Each sample was assayed in duplicate, and optical density at 450 nm (OD₄₅₀) was measured using a microplate reader.

To confirm coating efficiency, microwells coated with rhIFN‑γ or rhIL‑17A were probed using 0.1 µg/mL mouse anti‑human IFN‑γ antibody (unlabeled B27 mAb) or mouse anti‑human IL‑17A antibody (Sinobiological, Beijing, China), respectively. Bound antibodies were detected using HRP‑conjugated goat anti‑mouse immunoglobulins (1:3,000 dilution; SeraCare, MA, USA). The reaction was developed with TMB chromogen substrate for 10 minutes and stopped with 1 N HCl. OD₄₅₀ was measured using a microplate reader.

**Results**

To evaluate assay specificity, the developed ELISA was assessed for cross‑reactivity with rhIL‑17A. As shown in Fig 1, B27 mAb-HRP bound strongly to IFN‑γ but showed no detectable interaction with IL‑17A, confirming assay specificity. Likewise, plasma samples from both healthy controls (HC; n = 5) and AIGA⁺ individuals (n = 5) produced robust OD₄₅₀ signals in rhIFN‑γ–coated wells, whereas signals in rhIL‑17A–coated wells remained at background levels. These findings demonstrate that the assay selectively detects nAIGAs directed against IFN‑γ, without cross‑reactivity to unrelated cytokines. Importantly, antigen‑coated control wells yielded signals with both IFN‑γ and IL‑17A, thereby verifying the presence of coated antigen and confirming overall assay integrity.


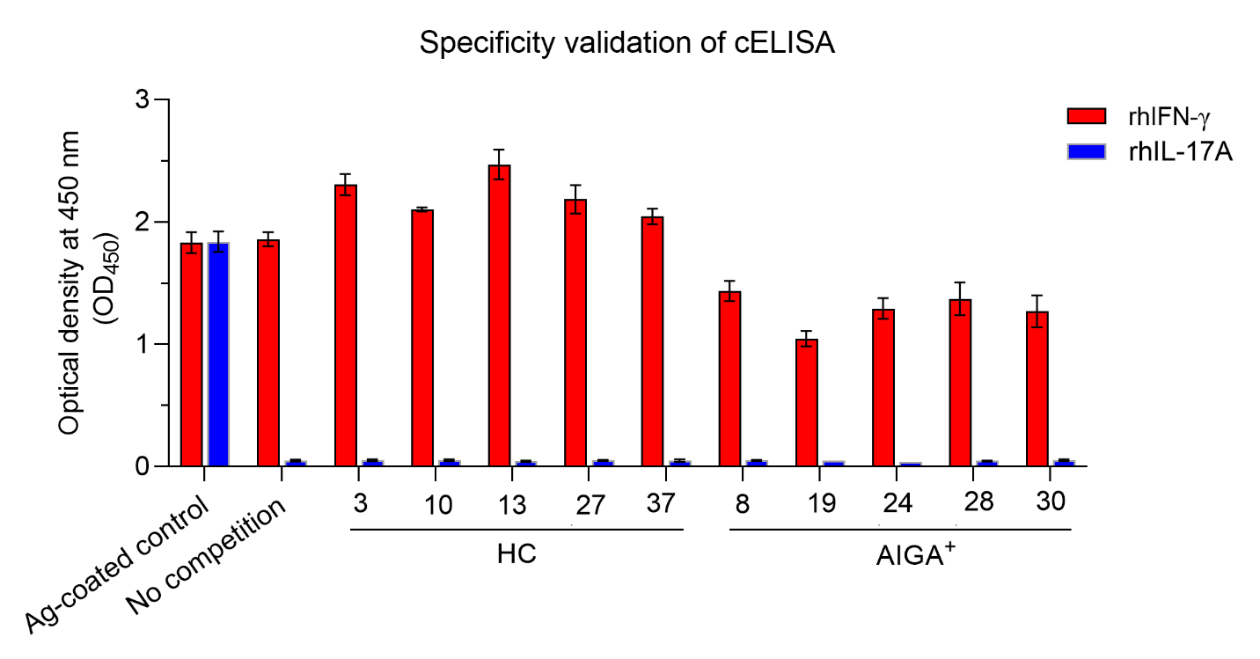


**Fig 1. Specificity validation of the developed cELISA.** Microwells were coated with rhIFN‑γ (red bars) or rhIL‑17A (blue bars), used as an irrelevant antigen. Representative plasma samples from healthy controls (HC; n = 5) and AIGA⁺ individuals (n = 5) were tested using the cELISA platform. Data are presented as mean ± SD of OD₄₅₀ values from duplicate wells. Ag‑coated control refers to antigen‑coated control wells; no competition indicates B27 mAb-HRP tested in the absence of plasma samples.
