# Supplementary material for: Development of a monoclonal antibody-based competitive ELISA as a surrogate assay for detecting neutralizing anti-interferon gamma autoantibodies in adult-onset immunodeficiency
Source: PLoS One. 2026 Mar 13;21(3):e0344451. doi: 10.1371/journal.pone.0344451 (PMC12987466; doi:10.1371/journal.pone.0344451)
Supplement: S1 Data — S2 Table. Dataset for sensitivity and specificity analysis of cell-based assay and cELISA using ROC analysis. S1 Fig. Flow cytometry gating strategy for determination of MHC class II expression in THP-1 cells. A representative gating strategy is shown for THP-1 cells under three conditions: no plasma, healthy control (HC), and AIGA-positive (AIGA⁺). THP-1 cells were first identified based on forward scatter height (FSC-H) and side scatter height (SSC-H) properties to exclude debris. Doublets were then removed by FSC-A versus FSC-H gating to define singlets. MHC class II–positive cells were subsequently identified based on FITC fluorescence intensity (Comp-FL1-H). The mean fluorescence intensity (MFI) of MHC class II–positive cells was used to calculate percentage inhibition. S1 Experiment. Assay specificity validation of cELISA. S1 Text. Assay performance of indirect ELISA. (ZIP) [file pone.0344451.s001.zip › Supporting Information/S1 Text.docx]

**S1 Text.** **Assay performance of indirect ELISA**.

**Results**

The performance of indirect ELISA for detecting AIGAs in plasma samples was evaluated using ROC analysis (Fig. 1). The ROC datasets for each assay are summarized in Table 1. The indirect ELISA yielded an area under the curve (AUC) of 1.000 (95% CI: 0.932–1.000, P < 0.0001), indicating excellent diagnostic accuracy (Fig. 1A). ROC analysis identified the optimal cut-off at OD₄₅₀ = 0.473, yielding 100% sensitivity and specificity with Youden’s index (J) of 1.00 (Fig. 1B).


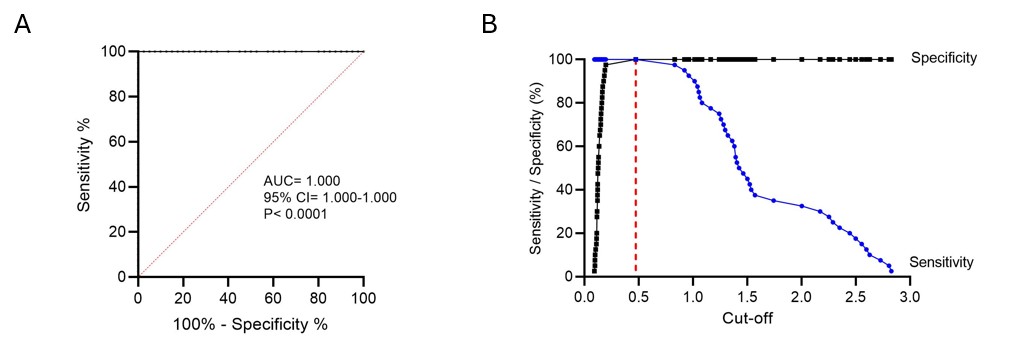


**Fig. 1. ROC analysis of indirect ELISA for detecting AIGAs.** ROC analysis was performed to evaluate the diagnostic performance of indirect ELISA. (A) The AUC provides a measure of diagnostic accuracy, with values between 0.800 and 1.000 generally considered indicative of good to excellent performance. (B) Sensitivity and specificity were plotted against cut-off values to determine the optimal threshold for indirect ELISA. The red dashed line marks the optimal cut-off that maximizes both sensitivity and specificity.

**Table 1.** Sensitivity and specificity at each cut-off value for indirect ELISA

| **Cut-off value** | **Sensitivity (%)** | **95% CI** | **Specificity (%)** | **95% CI** | **Likelihood ratio** |
| --- | --- | --- | --- | --- | --- |
| > 0.09150 | 100.0 | 91.24% to 100.0% | 2.500 | 0.1282% to 12.88% | 1.026 |
| > 0.09550 | 100.0 | 91.24% to 100.0% | 5.000 | 0.8884% to 16.50% | 1.053 |
| > 0.09650 | 100.0 | 91.24% to 100.0% | 7.500 | 2.584% to 19.86% | 1.081 |
| > 0.09750 | 100.0 | 91.24% to 100.0% | 10.00 | 3.958% to 23.05% | 1.111 |
| > 0.1025 | 100.0 | 91.24% to 100.0% | 12.50 | 5.460% to 26.11% | 1.143 |
| > 0.1090 | 100.0 | 91.24% to 100.0% | 15.00 | 7.061% to 29.07% | 1.176 |
| > 0.1115 | 100.0 | 91.24% to 100.0% | 17.50 | 8.745% to 31.95% | 1.212 |
| > 0.1125 | 100.0 | 91.24% to 100.0% | 20.00 | 10.50% to 34.76% | 1.250 |
| > 0.1135 | 100.0 | 91.24% to 100.0% | 27.50 | 16.11% to 42.83% | 1.379 |
| > 0.1160 | 100.0 | 91.24% to 100.0% | 30.00 | 18.07% to 45.43% | 1.429 |
| > 0.1185 | 100.0 | 91.24% to 100.0% | 35.00 | 22.13% to 50.49% | 1.538 |
| > 0.1195 | 100.0 | 91.24% to 100.0% | 37.50 | 24.22% to 52.97% | 1.600 |
| > 0.1205 | 100.0 | 91.24% to 100.0% | 40.00 | 26.35% to 55.40% | 1.667 |
| > 0.1215 | 100.0 | 91.24% to 100.0% | 42.50 | 28.51% to 57.80% | 1.739 |
| > 0.1230 | 100.0 | 91.24% to 100.0% | 47.50 | 32.94% to 62.50% | 1.905 |
| > 0.1255 | 100.0 | 91.24% to 100.0% | 50.00 | 35.20% to 64.80% | 2.000 |
| > 0.1275 | 100.0 | 91.24% to 100.0% | 52.50 | 37.50% to 67.06% | 2.105 |
| > 0.1285 | 100.0 | 91.24% to 100.0% | 55.00 | 39.83% to 69.29% | 2.222 |
| > 0.1340 | 100.0 | 91.24% to 100.0% | 60.00 | 44.60% to 73.65% | 2.500 |
| > 0.1395 | 100.0 | 91.24% to 100.0% | 65.00 | 49.51% to 77.87% | 2.857 |
| > 0.1440 | 100.0 | 91.24% to 100.0% | 67.50 | 52.02% to 79.92% | 3.077 |
| > 0.1485 | 100.0 | 91.24% to 100.0% | 70.00 | 54.57% to 81.93% | 3.333 |
| > 0.1500 | 100.0 | 91.24% to 100.0% | 72.50 | 57.17% to 83.89% | 3.636 |
| > 0.1520 | 100.0 | 91.24% to 100.0% | 75.00 | 59.81% to 85.81% | 4.000 |
| > 0.1550 | 100.0 | 91.24% to 100.0% | 77.50 | 62.50% to 87.68% | 4.444 |
| > 0.1595 | 100.0 | 91.24% to 100.0% | 80.00 | 65.24% to 89.50% | 5.000 |
| > 0.1625 | 100.0 | 91.24% to 100.0% | 82.50 | 68.05% to 91.25% | 5.714 |
| > 0.1635 | 100.0 | 91.24% to 100.0% | 85.00 | 70.93% to 92.94% | 6.667 |
| > 0.1670 | 100.0 | 91.24% to 100.0% | 87.50 | 73.89% to 94.54% | 8.000 |
| > 0.1760 | 100.0 | 91.24% to 100.0% | 90.00 | 76.95% to 96.04% | 10.00 |
| > 0.1825 | 100.0 | 91.24% to 100.0% | 92.50 | 80.14% to 97.42% | 13.33 |
| > 0.1870 | 100.0 | 91.24% to 100.0% | 95.00 | 83.50% to 99.11% | 20.00 |
| > 0.1960 | 100.0 | 91.24% to 100.0% | 97.50 | 87.12% to 99.87% | 40.00 |
| > 0.4725 | 100.0 | 91.24% to 100.0% | 100.0 | 91.24% to 100.0% |  |
| > 0.8310 | 97.50 | 87.12% to 99.87% | 100.0 | 91.24% to 100.0% |  |
| > 0.9205 | 95.00 | 83.50% to 99.11% | 100.0 | 91.24% to 100.0% |  |
| > 0.9610 | 92.50 | 80.14% to 97.42% | 100.0 | 91.24% to 100.0% |  |
| > 1.015 | 90.00 | 76.95% to 96.04% | 100.0 | 91.24% to 100.0% |  |
| > 1.040 | 87.50 | 73.89% to 94.54% | 100.0 | 91.24% to 100.0% |  |
| > 1.053 | 85.00 | 70.93% to 92.94% | 100.0 | 91.24% to 100.0% |  |
| > 1.063 | 82.50 | 68.05% to 91.25% | 100.0 | 91.24% to 100.0% |  |
| > 1.082 | 80.00 | 65.24% to 89.50% | 100.0 | 91.24% to 100.0% |  |
| > 1.163 | 77.50 | 62.50% to 87.68% | 100.0 | 91.24% to 100.0% |  |
| > 1.240 | 75.00 | 59.81% to 85.81% | 100.0 | 91.24% to 100.0% |  |

**Table 1.** Sensitivity and specificity at each cut-off value for indirect ELISA (Cont.)

| **Cut-off value** | **Sensitivity (%)** | **95% CI** | **Specificity (%)** | **95% CI** | **Likelihood ratio** |
| --- | --- | --- | --- | --- | --- |
| > 1.258 | 72.50 | 57.17% to 83.89% | 100.0 | 91.24% to 100.0% |  |
| > 1.281 | 70.00 | 54.57% to 81.93% | 100.0 | 91.24% to 100.0% |  |
| > 1.296 | 67.50 | 52.02% to 79.92% | 100.0 | 91.24% to 100.0% |  |
| > 1.320 | 65.00 | 49.51% to 77.87% | 100.0 | 91.24% to 100.0% |  |
| > 1.360 | 62.50 | 47.03% to 75.78% | 100.0 | 91.24% to 100.0% |  |
| > 1.381 | 60.00 | 44.60% to 73.65% | 100.0 | 91.24% to 100.0% |  |
| > 1.393 | 55.00 | 39.83% to 69.29% | 100.0 | 91.24% to 100.0% |  |
| > 1.404 | 52.50 | 37.50% to 67.06% | 100.0 | 91.24% to 100.0% |  |
| > 1.424 | 50.00 | 35.20% to 64.80% | 100.0 | 91.24% to 100.0% |  |
| > 1.463 | 47.50 | 32.94% to 62.50% | 100.0 | 91.24% to 100.0% |  |
| > 1.501 | 45.00 | 30.71% to 60.17% | 100.0 | 91.24% to 100.0% |  |
| > 1.522 | 42.50 | 28.51% to 57.80% | 100.0 | 91.24% to 100.0% |  |
| > 1.536 | 40.00 | 26.35% to 55.40% | 100.0 | 91.24% to 100.0% |  |
| > 1.570 | 37.50 | 24.22% to 52.97% | 100.0 | 91.24% to 100.0% |  |
| > 1.743 | 35.00 | 22.13% to 50.49% | 100.0 | 91.24% to 100.0% |  |
| > 2.002 | 32.50 | 20.08% to 47.98% | 100.0 | 91.24% to 100.0% |  |
| > 2.171 | 30.00 | 18.07% to 45.43% | 100.0 | 91.24% to 100.0% |  |
| > 2.253 | 27.50 | 16.11% to 42.83% | 100.0 | 91.24% to 100.0% |  |
| > 2.289 | 25.00 | 14.19% to 40.19% | 100.0 | 91.24% to 100.0% |  |
| > 2.351 | 22.50 | 12.32% to 37.50% | 100.0 | 91.24% to 100.0% |  |
| > 2.445 | 20.00 | 10.50% to 34.76% | 100.0 | 91.24% to 100.0% |  |
| > 2.499 | 17.50 | 8.745% to 31.95% | 100.0 | 91.24% to 100.0% |  |
| > 2.554 | 15.00 | 7.061% to 29.07% | 100.0 | 91.24% to 100.0% |  |
| > 2.598 | 12.50 | 5.460% to 26.11% | 100.0 | 91.24% to 100.0% |  |
| > 2.628 | 10.00 | 3.958% to 23.05% | 100.0 | 91.24% to 100.0% |  |
| > 2.727 | 7.500 | 2.584% to 19.86% | 100.0 | 91.24% to 100.0% |  |
| > 2.806 | 5.000 | 0.8884% to 16.50% | 100.0 | 91.24% to 100.0% |  |
| > 2.828 | 2.500 | 0.1282% to 12.88% | 100.0 | 91.24% to 100.0% |  |
