# Supplementary figures and images for: Development of a monoclonal antibody-based competitive ELISA as a surrogate assay for detecting neutralizing anti-interferon gamma autoantibodies in adult-onset immunodeficiency
Source: PLoS One. 2026 Mar 13;21(3):e0344451. doi: 10.1371/journal.pone.0344451 (PMC12987466; doi:10.1371/journal.pone.0344451)

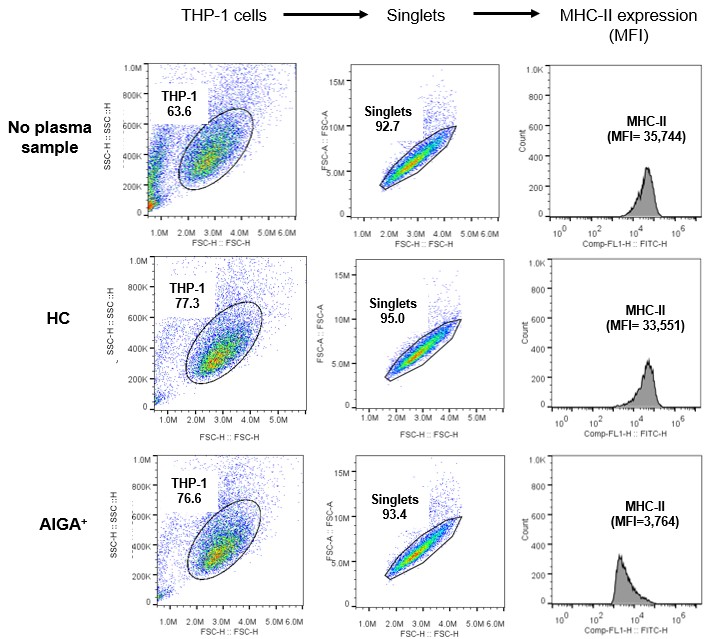

Supplement: S1 Data — S2 Table. Dataset for sensitivity and specificity analysis of cell-based assay and cELISA using ROC analysis. S1 Fig. Flow cytometry gating strategy for determination of MHC class II expression in THP-1 cells. A representative gating strategy is shown for THP-1 cells under three conditions: no plasma, healthy control (HC), and AIGA-positive (AIGA⁺). THP-1 cells were first identified based on forward scatter height (FSC-H) and side scatter height (SSC-H) properties to exclude debris. Doublets were then removed by FSC-A versus FSC-H gating to define singlets. MHC class II–positive cells were subsequently identified based on FITC fluorescence intensity (Comp-FL1-H). The mean fluorescence intensity (MFI) of MHC class II–positive cells was used to calculate percentage inhibition. S1 Experiment. Assay specificity validation of cELISA. S1 Text. Assay performance of indirect ELISA. (ZIP) [file pone.0344451.s001.zip › Supporting Information/S1 Fig.jpg]
